# A Metabolomics study of metabolites associated with the glomerular filtration rate

**DOI:** 10.1186/s12882-023-03147-9

**Published:** 2023-04-21

**Authors:** Hongquan Peng, Xun Liu, Chiwa Ao Ieong, Tou Tou, Tsungyang Tsai, Haibin Zhu, Zhi Liu, Peijia Liu

**Affiliations:** 1 Department of Nephrology, Kiang Wu Hospital, Macau, China; 2 Department of Nephrology, The Third Affiliated Hospital of Sun Yat-sen University, Guang Zhou, China; 3 Department of Statistics and Data Science, School of Economics, Jinan University, Guangzhou, China; 4 Department of Mathematics, University of Macau, Macau, China

**Keywords:** Chronic kidney disease (CKD), Glomerular filtration rate (GFR), Biomarker, Metabolomics, Metabolite

## Abstract

**Background:**

Chronic kidney disease (CKD) is a global public health issue. The diagnosis of CKD would be considerably enhanced by discovering novel biomarkers used to determine the glomerular filtration rate (GFR). Small molecule metabolites related to kidney filtration function that might be utilized as biomarkers to measure GFR more accurately could be found via a metabolomics analysis of blood samples taken from individuals with varied glomerular filtration rates.

**Methods:**

An untargeted metabolomics study of 145 plasma samples was performed using ultrahigh-performance liquid chromatography tandem mass spectrometry (UPLC–MS/MS). The 145 samples were divided into four groups based on the patient’s measured glomerular filtration rates (mGFRs) determined by the iohexol plasma clearance rate. The data were analyzed using random forest analyses and six other unique statistical analyses. Principal component analysis (PCA) was conducted using R software.

**Results:**

A large number of metabolites involved in various metabolic pathways changed significantly between groups with different GFRs. These included metabolites involved in tryptophan or pyrimidine metabolism. The top 30 metabolites that best distinguished between the four groups in a random forest plot analysis included 13 amino acids, 9 nucleotides, and 3 carbohydrates. A panel of metabolites (including hydroxyaparagine, pseudouridine, C-glycosyltryptophan, erythronate, N-acetylalanine, and 7-methylguanidine) for estimating GFR was selected for future testing in targeted analyses by combining the candidate lists with the six other statistical analyses. Both hydroxyasparagine and N,N-dimethyl-proline-proline are unique biomarkers shown to be inversely associated with kidney function that have not been reported previously. In contrast, 1,5-anhydroglucitol (1,5-AG) decreases with impaired renal function.

**Conclusions:**

This global untargeted metabolomics study of plasma samples from patients with different degrees of renal function identified potential metabolite biomarkers related to kidney filtration. These novel potential metabolites provide more insight into the underlying pathophysiologic processes that may contribute to the progression of CKD, lead to improvements in the estimation of GFR and provide potential therapeutic targets to improve kidney function.

## Introduction

Chronic kidney disease (CKD) significantly increases the risk of cardiovascular mortality and morbidity and has become a severe public health issue, affecting 10% of the worldwide population. [1–3]. Accurate assessment of the glomerular filtration rate (GFR) is vitally important for evaluating renal function in clinical practice. There are two kinds of GFR assessments. The first is the measured glomerular filtration rate (mGFR) obtained by measuring the clearance rate of exogenous filtration markers such as inulin and iohexol and is regarded as the reference standard (i.e., gold standard) [4, 5]. The second is the estimated glomerular filtration rate (eGFR), calculated by measuring endogenous markers, such as creatinine [6]. Unfortunately, measuring the clearance rate of exogenous filtration markers such as inulin and iohexol are both costly and cumbersome. Therefore, GFR is often estimated in clinical practice using the serum creatinine concentration. In this study, mGFR was based on plasma iohexol clearance, while eGFR was based on the well-known chronic kidney disease epidemiology collaboration (CKD-EPI) equations for estimating GFR. Creatinine is a well-established clinical biomarker for assessing kidney function [7], although it has sensitivity issues that do not warrant its application in the early detection of CKD [8, 9]. Creatinine levels rise only after almost half of kidney function has been lost and are also dependent on other factors, such as ethnicity and differences in muscle mass.

Biomarkers that can improve the accuracy of eGFR and help clinicians to make a timely and appropriate diagnosis and treatment of CKD are in great demand. In addition to using the creatinine concentration to calculate eGFR, adding cystatin C to the eGFR calculation is known to improve eGFR accuracy and is a better predictor for the future risk of end-stage renal disease (ESRD) and death [10]. However, novel biomarkers are still needed to further improve the precision of eGFR calculations. This would also reduce estimation errors caused by variation in non-GFR determinants of each marker, in addition to avoiding the use of ethnicity and clinical characteristics as surrogates for non-GFR determinants [11, 12].

It is well established that renal function affects blood metabolite concentrations, and therefore, it is likely that some metabolites may be useful for estimating kidney function. Metabolomics is an omics technology that identifies and quantitatively compares all small molecule metabolites among different groups of biological samples and is therefore an ideal technique for identifying unknown blood metabolite markers potentially associated with impaired kidney function. Several untargeted and targeted metabolomics studies have been conducted, and a few new biomarkers have been reported [13–16]. The objective of our study was to discover novel glomerular filtration-related blood metabolite biomarkers using untargeted metabolomics analysis in a Chinese population. In this study, 145 plasma samples from CKD patients and healthy volunteers were divided into four groups based on mGFR determined by the iohexol clearance rate [17–19]. Untargeted metabolomics analysis was then performed to identify differentially changed plasma metabolites among the four groups. The study was designed to identify blood metabolites associated with mGFR and CKD progression and select candidate markers that improved the estimation of eGFR.

## Materials and methods

### Study participants and sample collection

A total of 145 individuals (including 10 healthy volunteers) with varying degrees of renal function were enrolled in this study carried out at Kiang Wu Hospital in 2019. The study protocol was approved by the local ethics committee. Signed, informed consent forms were obtained from each participant. Plasma samples were collected from each participant, and the measured GFR (mGFR) of each sample was determined by iohexol plasma clearance. The diagnosis of CKD was based on the NKF-K/DOQI guidelines and was defined as kidney damage or a glomerular filtration rate (GFR) < 60 mL/min/1.73 m^2^ for 3 months or longer, irrespective of cause.

The inclusion criteria were as follows: (1) age > 18 years and (2) healthy volunteers and voluntary CKD patients. The exclusion criteria were as follows: (1) acute kidney injury; (2) congestive heart failure, obvious peripheral edema, dehydration and other severe fluid balance disorders; (3) skeletal muscle atrophy and physical disability; (4) urinary tract obstruction; and (5) patients who had recently taken the following medications but were unable to stop taking them, including those who had recently taken aspirin, nonsteroidal anti-inflammatory drugs, cimetidine, ranitidine, and others. ; (6) allergy to iodine contrast agents; (7) pregnancy or breastfeeding; (8) presence of thyroid disease; and (9) dialysis. and (10) cancer patients.

The mGFR to evaluate kidney function was obtained by measuring the plasma clearance of iohexol [19]. The participants were tested in the non-fasting state with each receiving a single infusion of 5 ml of iohexol (300 mg/mL, GE Healthcare, Shanghai, China). Blood samples were drawn from the other arm. Plasma samples were collected, aliquoted, and stored at -80 °C until analysis. An untargeted ultra-performance liquid chromatography-tandem mass spectrometry (UPLC–MS/MS) metabolomics analysis was conducted on the collected plasma samples.

## Metabolomics Analysis

### Sample preparation

The untargeted metabolomics analysis was conducted at the Dian Calibra-Metabolon Joint Metabolomics Laboratory (Hangzhou, China). To detect different types of metabolites that cannot be successfully analyzed using a single LC‒MS method, four different UPLC–MS/MS assays were conducted for each plasma sample. Briefly, a methanol-based sample extraction solution was added to each plasma sample, and proteins in the plasma samples were precipitated with vigorous shaking in a GenoGrinder 2010 (Spex SamplePrep, USA) for two minutes, followed by centrifugation to pellet the proteins and other debris. The resulting supernatant containing extracted metabolites was collected and divided into four fractions: two fractions for reversed-phase (RP) UPLC–MS/MS analyses in positive ion electrospray ionization (ESI) mode in two different chromatography programs; one fraction for RP/UPLC–MS/MS analysis in negative ion ESI; and the last fraction for hydrophilic interaction chromatography (HILIC)/UPLC–MS/MS under negative ion ESI. Liquid transfer between sample plates were processed using a Hamilton automated MicroLab STAR system (Hamilton, Switzerland). Each fraction was dried under nitrogen gas flow to remove the organic solvent. Before injection into each corresponding UPLC–MS/MS system, the dried extract was redissolved in a reconstitution solution appropriate for each assay[20].

### UPLC‒MS/MS

**A** Waters ACQUITY 2D UPLC system and Thermo Fisher Scientific Q-Exactive (QE) high-resolution/accurate mass orbitrap spectrometer were used for UPLC‒MS/MS analysis. For the three UPLC methods using reversed-phase liquid chromatography, C_18_ columns (UPLC BEH C_18_-2.1 × 100 mm, 1.7 μm; Waters) were used. The UPLC column used in the fourth method was a HILIC column (UPLC BEH Amide 2.1 × 150 mm, 1.7 μm; Waters) for metabolites with strong polarity. The QE mass spectrometer analysis alternated between MS and data-dependent MS2 scans using dynamic exclusion at 35,000 mass resolution. The scan range was 70–1,000 m/z. A quality control sample was prepared by pooling a small fraction of each experimental sample together. This QC sample was inserted multiple times throughout the sample sequence and analyzed along with the experimental samples. A mixture of internal standards was spiked into every sample, and the IS signals were observed to monitor the UPLC‒MS/MS instrument stability.

#### Data processing and metabolite identification

The raw mass spectrometry data were processed and extracted, peaks were identified using software developed in-house, and metabolites were identified by comparison of experimental ion features to library entries of purified reference standards. The library entries include the retention time/index (RI), mass to charge ratio (m/z), and MS/MS spectral data of reference standards. A positive identification is based on three criteria: retention index within a narrow window of the proposed identification, accurate mass match to the library +/- 10 ppm, and the MS/MS forward and reverse scores between the experimental data and authentic standards [20]. The scan range of the mass spectrometer in the metabolomics experiment was set to 80 − 1,000 Daltons.

### Statistical methods

Principal component analysis (PCA) was conducted using R software. PCA is a dimension reduction technique that allows differences between a large number of variables to be represented by a smaller number of variables. The detected metabolites represent the variables in this situation. PCA permits visualization of how individuals within a group cluster with respect to their data-compressed principal components. This method can therefore help determine if samples segregate based on metabolite signature differences.

The data are expressed as the mean ± standard deviation (SD). Group comparisons were carried out using the chi-square or Mann–Whitney test, while the T test was used for comparisons between two groups. Mean values and proportions were compared using one-way analysis of variance and chi-square tests, respectively. SPSS-IBM22 was used for these analyses, with differences in all the tests considered significant if the *p* value was < 0.05. A random forest analysis was used to select the metabolites that had the largest contribution to the distinction between groups. In addition, we used six well-known feature selection statistical methods to further select the first 30 most important metabolites that explained changes in mGFR. These methods included the least absolute shrinkage and selection operator (LASSO), optimal least absolute shrinkage and selection operator (Opt-Lasso), smoothly clipped absolute deviations (SCAD), iterative sure independent screening (ISIS), robust rank correlation-based screening (RRCS), and partial least squares (PLS). These analyses were performed using RStudio [20, 21].

## Results

### Demographic and clinical characteristics of the participants

The mGFRs of the 145 samples were analyzed by the plasma clearance of iohexol. The 145 samples collected were divided into four groups based on the mGFR values (Table 1). The control group contained 37 samples with an mGFR > 90 ml/min/1.73m^2^, the mild kidney dysfunction group contained 39 samples with an mGFR 60 - <90 ml/min/1.73m^2^, the moderate nephropathy group contained 47 samples with an mGFR 30 - <60 ml/min/1.73m^2^, and the severe nephropathy group contained 22 samples with an mGFR < 30 ml/min/1.73m^2^.

**Table 1:** Demographic and clinical characteristics of the study population

Variable	mGFR ≥ 90 ml/min per 1.73 m^2^	60 ≤ mGFR<90 ml/min per 1.73 m^2^	30 ≤ mGFR<60 ml/min per 1.73 m^2^	mGFR<30 ml/min per 1.73 m^2^	over all	P
Sample size,	37	39	47	22	145	
Age, y	41.9(10.6)	60.7(14.3)	72.3(13.5)	74.9.(17.0)	61.8(18.6)	< 0.001
Male, %	16(43.2)	18(46.2)	25(53.2)	9(40.9)	68(43.9%)	0.736
Body mass index, kg/m^2^	23.6(13.7)	24.7(4.5)	24.5(5.2)	24.1.(3.0)	24.6(4.6)	0.357
Diabetes prevalence, %	4(10.8)	7(17.9)	11(23.4)	10(45.5))	33(22.1)	0.024
Hypertention prevalence, %	4(10.8)	15(38.4)	28(59.6)	15(68.2)	62/(42.7)	< 0.001
Hyperuricemia prevalence, %	4(10.8)	6(15.4)	10(21.3)	5(22.7)	25(17.2)	0.524
Coronary artery disease prevalence, %	2(5.4)	5(12.8)	11(23.4)	9(40.9)	27 (18.6)	0.046
Lipid-lowering medication use, %	2(5.4)	6(15.3)	5(10.6)	2(9.1)	15 (10.3)	0.314
Uric acid-lowing medication use, %	4(10.8)	6(15.4)	10(21.3)	5(22.7)	25(17.2)	0.524
Antiplatelet drug use, %	2(5.4)	5((12.8)	11(23.4)	9(40.9)	27 (18.6)	0.023
Antihypertensive medication use, %	4(10.8)	15(38.4)	28(59.6)	15(68.2)	62/(42.7)	< 0.001
glucose-lowering drug use, %	4 (10.8)	7(17.9)	11(23.4)	10(45.5))	33(22.1)	0.024
Systolic BP, mmHg	127.6(13.5)	135(17.3)	135.8(17.6)	136.5(20.2)	133.8(17.6)	0.213
Diastolic BP, mmHg	77.4(12.1)	79.8(12.6)	75.6(14.2)	78.3(14.5)	77.8(13.8)	0.354
Creatinine	0.82(0.4)	1.2(0.8)	1.9(2.1)	4.2(2.6)	1.85 (2.0)	< 0.001
Cystatin C	0.83(0.81)	1.3(0.6)	1.8(0.5)	3.2(1.3)	1.27(1.2)	< 0.001
eGFRcr-cys, ml/min per 1.73 m^2^	103.1(16.2)	75.5(23.2)	49.3(17.1)	23.2(12.1)	66.1(32.7)	< 0.001
mGFR, ml/min per 1.73 m^2^	106.8(12.3)	73.2(8.5)	44.6(7.8)	21.9(7.5)	64.73(30.3)	

Table 1. Continuous measures are summarized as the mean ± standard deviation (SD), and categorical variables are given as percentages. Values for categorical variables are given as numbers (percentages). Abbreviations: BP, blood pressure; eGFRcr-cys, estimated glomerular filtration rate, calculated by Chronic Kidney Disease Epidemiology Collaboration, mGFR, measured glomerular filtration rate. Hypertension was defined as systolic BP ≧ 90 mm Hg or 140 or diastolic BP ≧ 90 mm Hg or receiving antihypertensive medications. Diabetes was defined as fasting blood glucose ≧ 126 mg/dL or receiving antidiabetic medications. Hyperuricemia was defined as uric acid levels ≧ 6 mg/dL (female) and ≧ 7.0 mg/dL (male) or receiving uric acid-lowering medication

Table 1 shows the demographic and clinical information of the 145 participants, 68 (46.9%) of whom were males. The mean age was 61.81 ± 18.59 years (range 20–96 years), mean body mass index (BMI) was 24.2 ± 4.2 kg/m^2^ (range 15.0-48.6 kg/ m^2^), mean serum creatinine was 1.82 ± 2.01 g/L (range 0.42–10.79 mg/L), and mean cystatin C concentration was 1.17 ± 1.16 mg/L (range 0.62–5.82 mg/L). There was no obvious difference in the sex ratio, BMI, or blood pressure between the four groups, although the individuals in the severe nephropathy group tended to be older (mean age 74.9 years). The group with impaired renal function had a higher prevalence of comorbidities (hypertension, diabetes, and coronary artery disease) than the group with normal kidney function.

### Global metabolite changes associated with varying levels of mGFR

The untargeted metabolomics analysis identified a total of 1094 compounds with known biochemical identities. ANOVA contrasts were used to identify metabolites that differed significantly between the four groups. A summary of the numbers of metabolites that achieved statistical significance (p ≤ 0.05), as well as those approaching significance (0.05 < p < 0.10), is shown below. A large number of metabolites changed significantly when mGFR decreased. For example, when comparing the severe nephropathy group with the normal control group, 60% of the detected metabolites (659 of 1094) changed significantly (*p* < 0.05) (Table 2).

**Table 2:** The number of significantly changed metabolites among different groups

Anova Contrasts	Mild	Moderate	Severe	Moderate	Severe	Severe
	Normal	Normal	Normal	Mild	Mild	Moderate
**Total metabolites**	217	531	659	309	578	476
**Metabolites**(↑↓) (p < 0.05)	**133/84**	**341/190**	**455/204**	**228/81**	**421/157**	**377/99**
**Total metabolites**	**73**	**72**	**49**	**94**	**44**	**54**
**Metabolites**(↑↓) (0.05 < p < 0.10)	**41/32**	**43/29**	**27/22**	**64/30**	**19/25**	**35/19**

### High level of metabolite overview

**Fig. 1:**
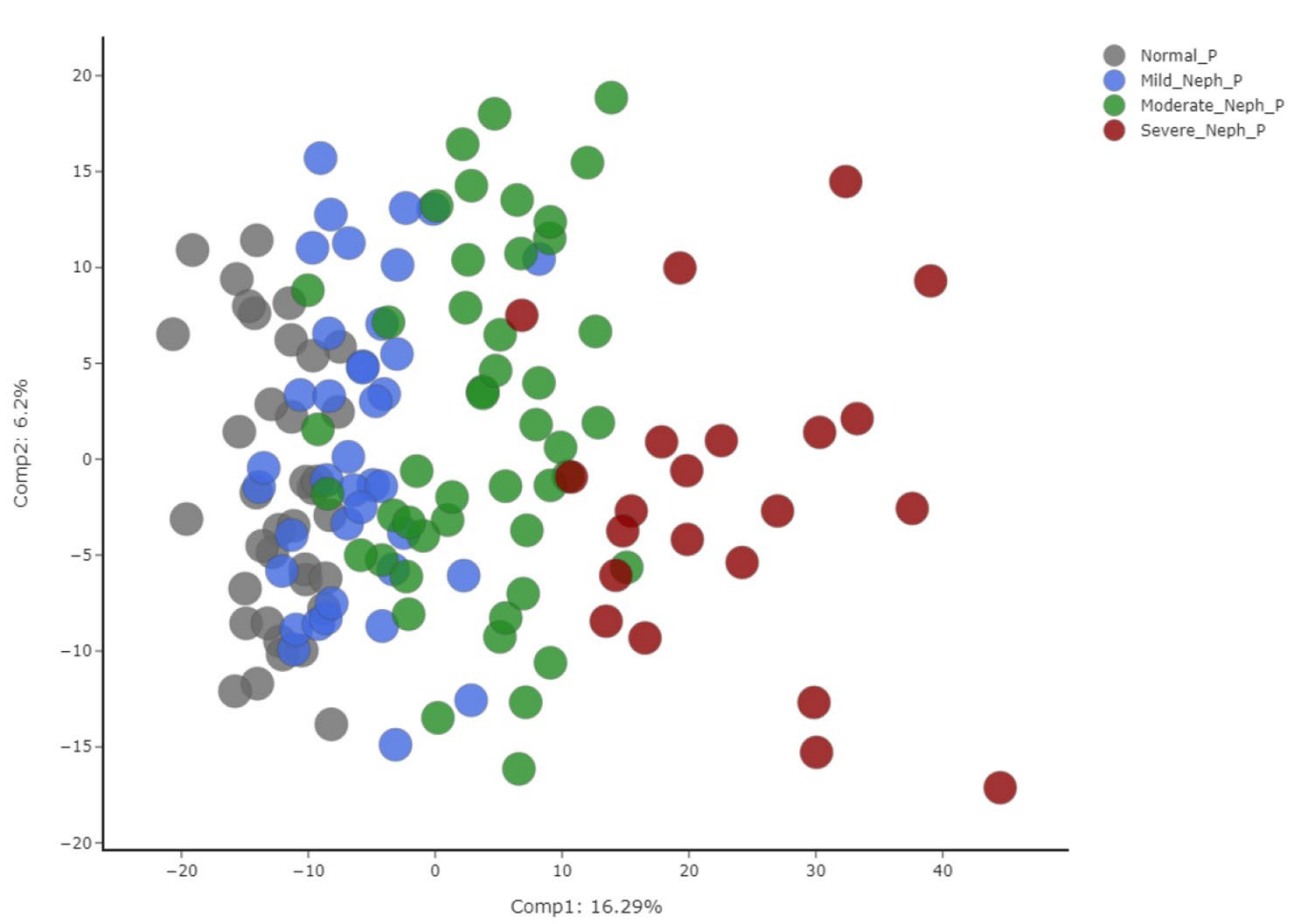
PCA of *plasma* from subjects with normal kidney function (gray) and subjects with mild (blue), moderate (green), and severe (red) nephropathy

As shown in Fig. 1, the PCA of samples were colored by sample type. There was a clear separation between the sample types, generally along the PC1 axis, with samples from subjects with normal kidney function on the left, severe nephropathy on the right, and mild and moderate nephropathy in the middle. This indicates that there were significant differences in phenotype between the four groups.

### Identification of the top ranking metabolites associated with kidney function

**Fig. 2:**
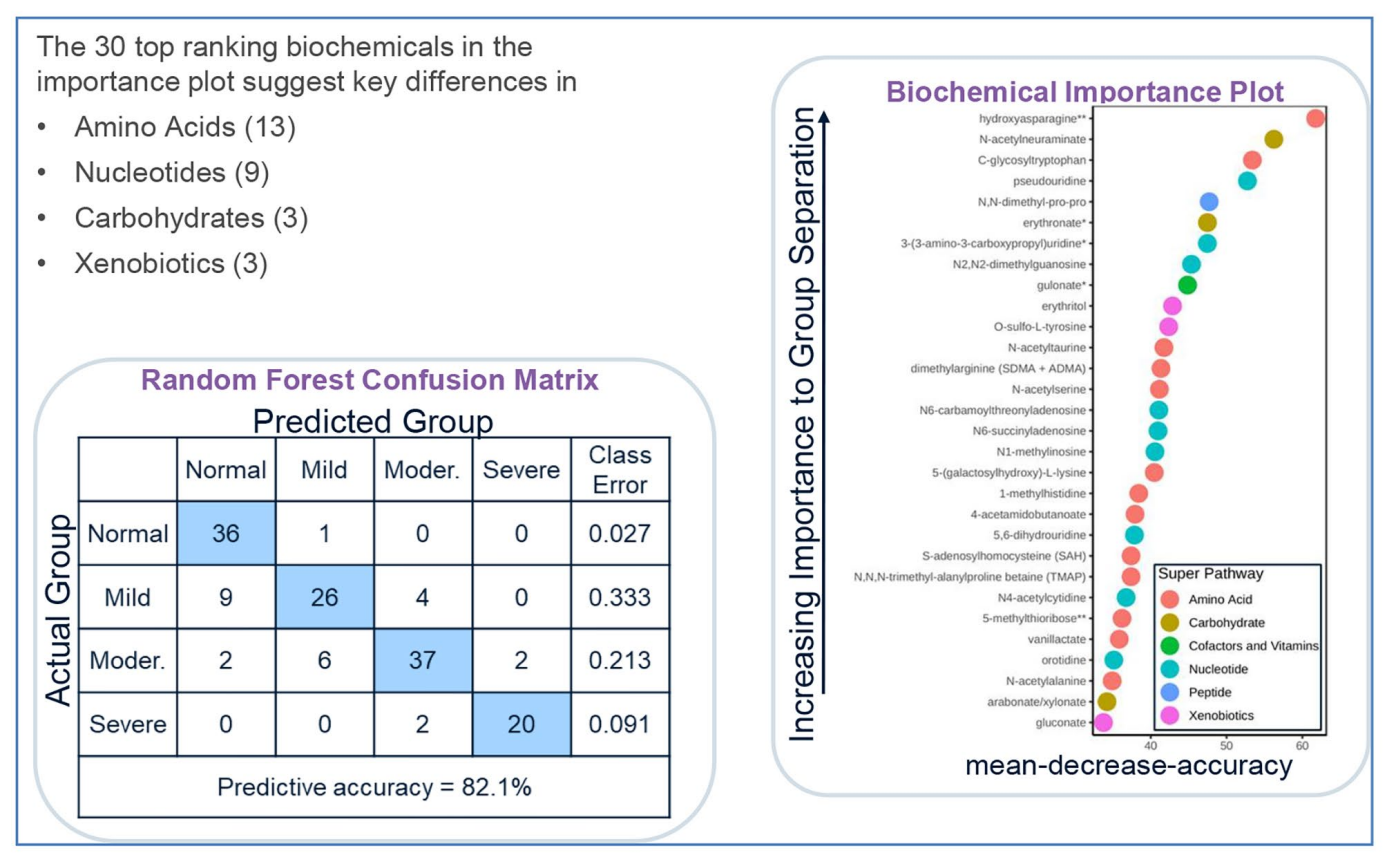
Random forest analysis of plasma from subjects with normal kidney function, mild nephropathy, moderate nephropathy, and severe nephropathy

This analysis achieved a predictive accuracy of 82.1% compared to 25% by random chance alone, confirming that there were significant metabolic differences between the four groups. As shown in Fig. 2, the top 30 metabolites that contributed the most to distinctions between the four groups included 13 amino acids (hydroxyasparagine, C-mannosyltryptophan, N-acetyltaurine, dimethylarginine [SDMA + ADMA], N-acetylserinenine, 5-[galactosylhydroxy]-L-lysine, 1-methylhistidine, 4-acetamidobutanoate, S-adenosylhomocysteine [SAH], N,N,N-trimethyl-alanylproline betaine [TMAP], 5-methylthioribose, vanillactate, and N-acetylalanine), nine nucleotides (pseudouridine, 3-[3–amino-3-carboxypropyl] uridine, N2,N2-dimethylguanosine, N6-carbamoylthreonyladenosine, N6-succinyladenosine, N1-methyladenosine, 5,6-dihydrothymine, N4-acetylcytidine, and orotidine), and three carbohydrates (N-acetylneuraminate, erythronate, and arabonate/xylonate). The direct correlations between the level of the top 10 metabolites and mGFR are shown in Table 3.

**Table 3:** Correlations between the levels of the top ten metabolites and measured GFR

Variable	mGFR	
	r	P value
1.Hydroxyasparagine	-0.739	< 0.001***
2.N-acetylneuraminate	-0.689	< 0.001***
3.C-glycosyltryptophan	-0.751	< 0.001***
4.Pseudouridine	-0.723	< 0.001***
5.N,N-dimethyl-proline-proline	-0.625	< 0.001***
6.Erythronate	-0.640	< 0.001***
7.3-(3-amino-3-carboxypropyl)uridine	-0.711	< 0.001***
8.N2,N2-dimethylguanosine	-0.683	< 0.001***
9.gulonate	-0.644	< 0.001***
10.erythritol	-0.482	< 0.001***

R for Pearson correlation coefficients (***P < 0.001).

**Fig. 3:**
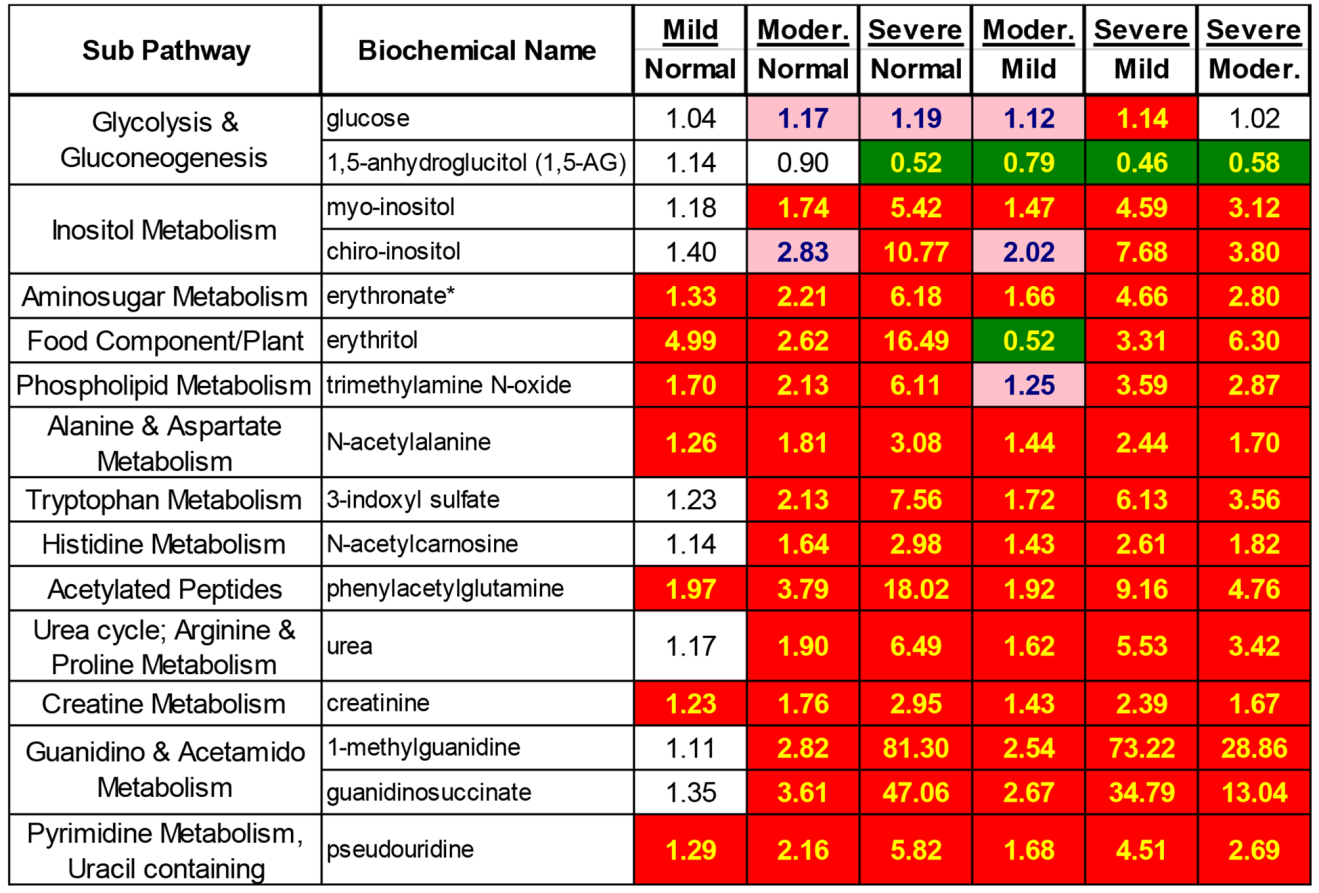
Significantly changed metabolites involved in kidney function

**Fig. 4:**
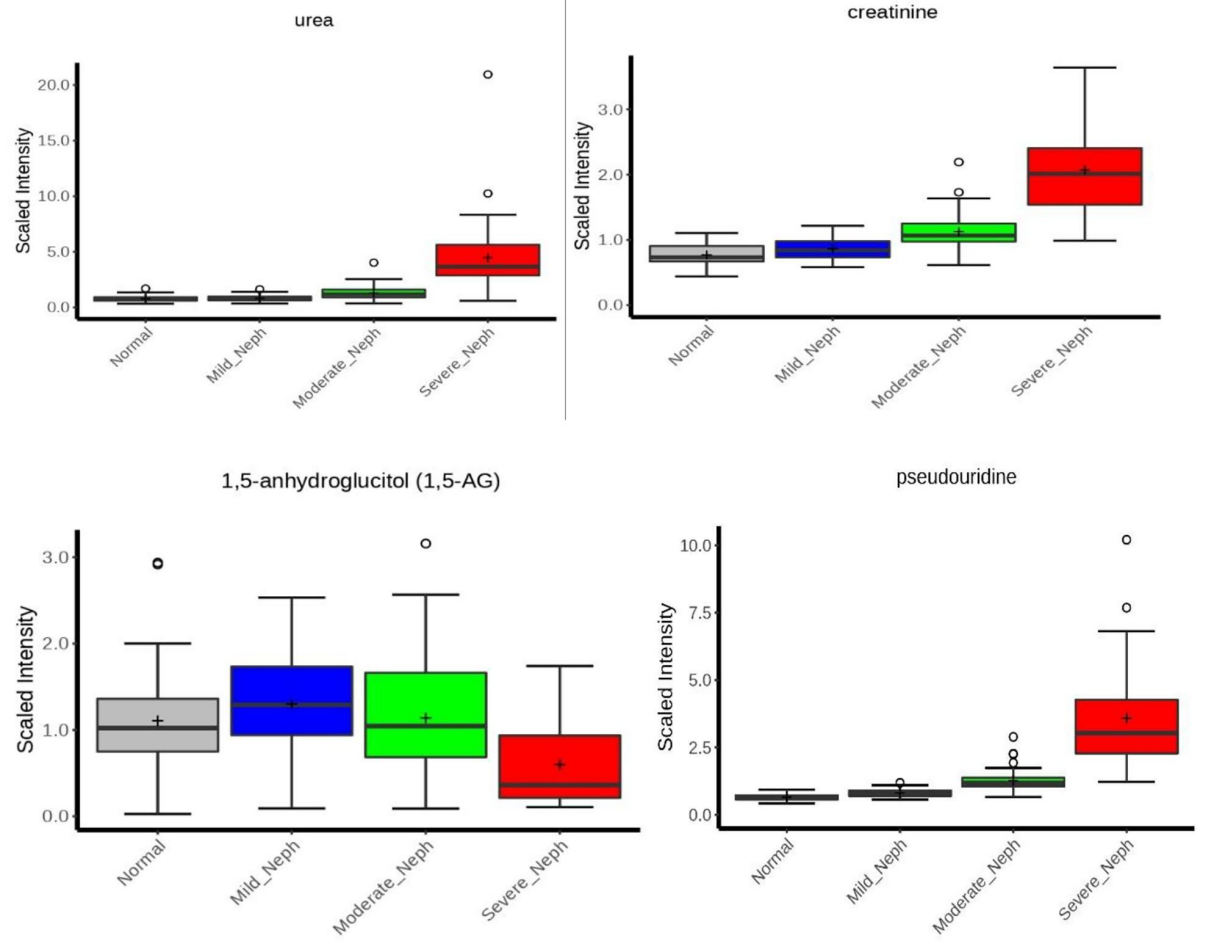
Urea, creatinine, 1,5-anhydroglucitol and pseudouridine are displayed in the plots located in the accompanying box plot files

In addition to the traditional clinical markers of kidney dysfunction, such as increased creatinine and urea (Figs. 3 and 4), many other metabolites reported to be related to kidney function were also shown to be increased significantly with decreased mGFR. As one function of the kidney is to remove toxins and excess metabolites from the body, kidney dysfunction can result in the buildup of many endogenous and exogenous metabolites. These included erythronate, myo-inositol, chiro-inositol, trimethylamine oxide, 3-indoxyl sulfate, phenylacetylglutamine, 1-methylguanidine, guanidinosuccinate, N-acetylalanine, N-acetylcarnosine, and pseudouridine, which also showed increasing levels with increased nephropathy (Fig. 3).

**Fig. 5:**
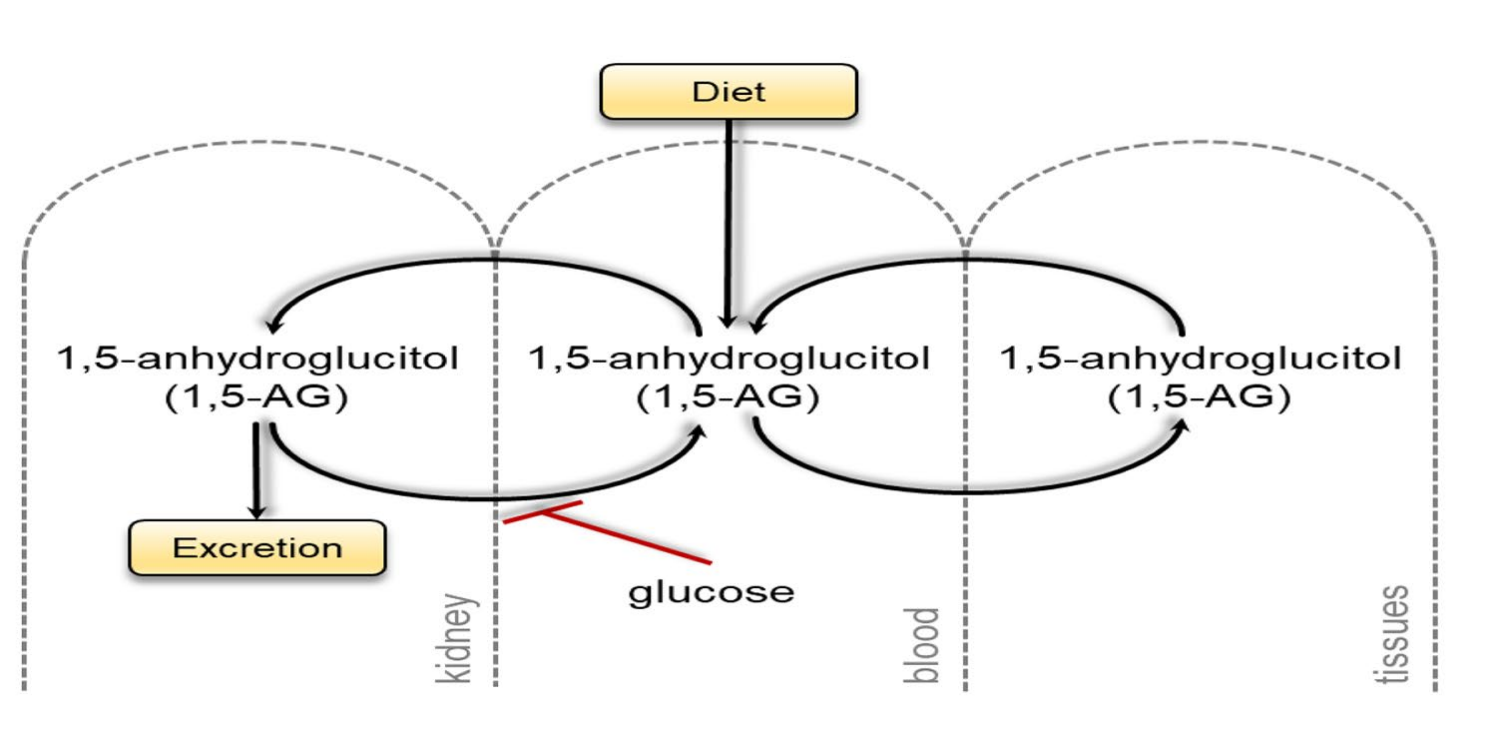
Metabolism of 1,5-anhydroglucitol

In contrast, 1,5-anhydroglucitol (1,5-AG) decreased with impaired renal function (Figs. 3 and 4). In the kidney, 1,5-AG is filtered by the glomerulus, and the majority is reabsorbed in the proximal tubule and returned to the blood. Glucose, which is trending higher in the moderate and severe nephropathy groups compared to the normal kidney function group, is a competitive inhibitor of this reabsorption. Thus, if glucose rises, more 1,5-AG is excreted in the urine, lowering blood levels (Fig. 5).

### Six statistical methods for model selection

**Fig. 6:**
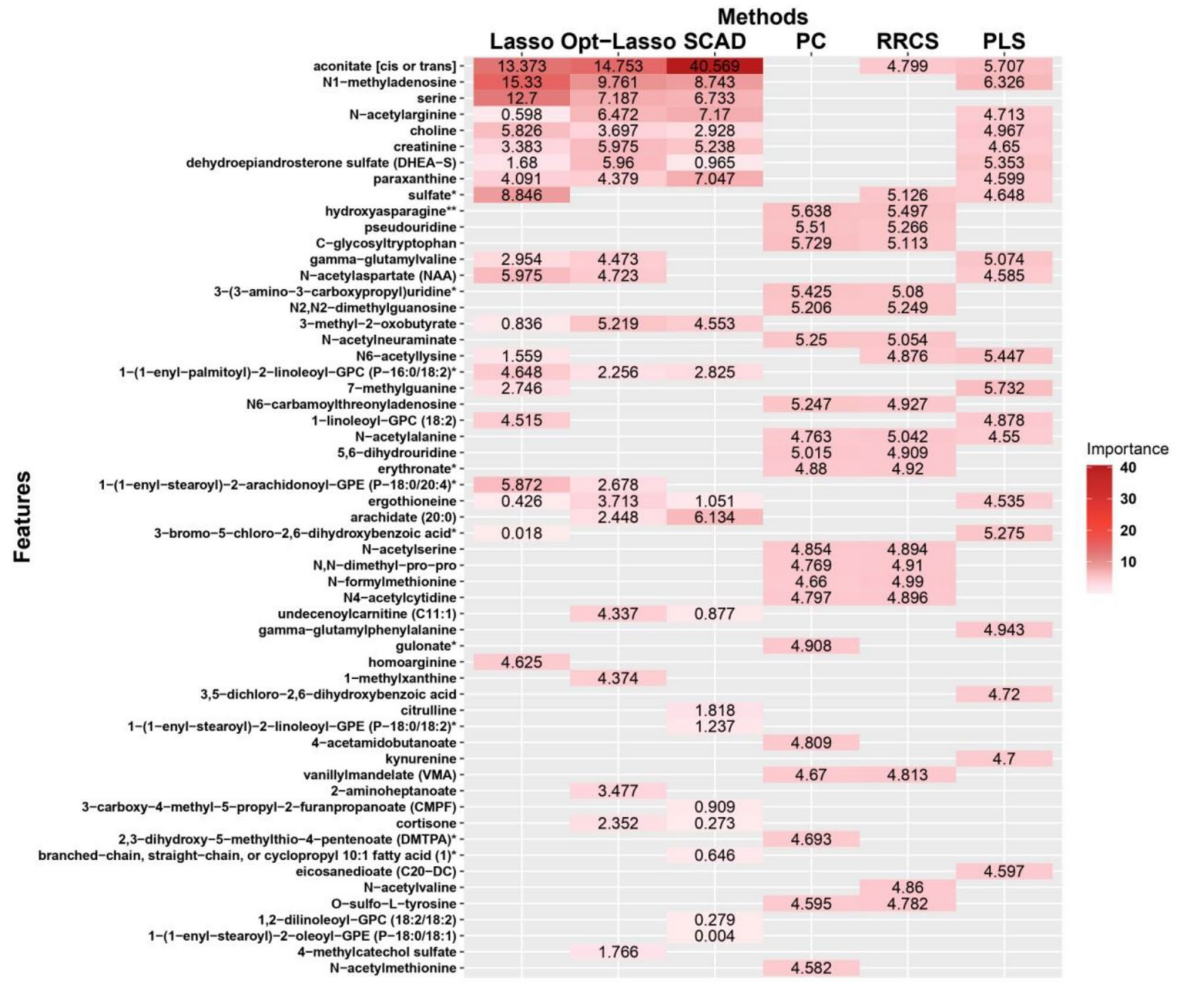
Feature selection using 6 statistical methodologies in the form of a heat plot, including the least absolute shrinkage and selection operator (LASSO), optimal least absolute shrinkage and selection operator (Opt-Lasso), smoothly clipped absolute deviations (SCAD), iterative sure independent screening (ISIS), robust rank correlation-based screening (RRCS), and partial least squares (PLS).

In addition to the random forest analysis, we used six other feature screening methods to rank the importance of the 1094 indices of metabolites in the dataset of 145 participants. In fact, because each of the other six statistical screening approaches had its own benefits and drawbacks, it was challenging to determine which model was the best. A suitable approach was to apply all of them while attempting to attain balance, without taking into account any additional mathematical criteria. Therefore, we screened and validated the variables identified by the random forest analysis using the six statistical approaches. Since the screening methods heavily depend on mathematical conditions, we can see that the results obtained with different methods are quite different. Nevertheless, we can still see that the important features identified by most screening methods have many overlaps with the important variables in the random forest. This showed that creatinine was one of the most important metabolites and was sixth in the overall ranking calculated by taking a weighted average of the results of all six methods. In addition to creatinine, several other metabolites were found to be strongly correlated with kidney function. Ranked from the highest down, these metabolites were aconitate (cis or trans), N1 − methyladenosine, serine, N − acetylarginine, choline, creatinine, dehydroepiandrosterone sulfate (DHEA − S), paraxanthine, sulfate, hydroxyasparagine, pseudouridine, and C − glycosyltryptophan (Fig. 6).

## Discussion

The kidney filters waste products from the blood to be excreted in the urine, with filtration functionality usually evaluated by determining the GFR. To obtain a more accurate assessment of eGFR, it is necessary to discover new biomarkers that can be incorporated into the equation used to calculate the index. Recent advances in mass spectrometry methodology have allowed comprehensive studies of metabolomes and their relationship with kidney function [20, 22–26]. Metabolomics studies can identify and quantify all metabolites present in a given sample, covering hundreds to thousands of metabolites. In the current study, we performed an untargeted metabolomics study to identify some novel metabolite markers that showed significant changes between groups with different mGFRs. Sekula et al. [27] reported 56 metabolites that were associated with eGFR determined using creatinine (eGFRcr), of which the top six metabolites were pseudouridine, c-mannosyltryptophan, N-acetylalanine, erythronate, myo-inositol, and N-acetylcarnosine. Coresh et al. [13] also reported that pseudouridine, acetylthreonine, myoinositol, phenylacetylglutamine, and tryptophan showed a strong correlation with mGFR.

In our study, many of these reported metabolites associated closely with GFR were also identified and changed significantly in the four sample groups. These metabolites included C-glycosyltryptophan (also known as C-mannosyltryptophan), pseudouridine, N-acetylalanine, erythronate, myo-inositol, and N-acetylcarnosine. In the severe nephropathy group, N-acetylalanine increased 2.7-fold, N-acetylcarnosine increased 1.8-fold, and pseudouridine increased 4.5-fold compared to their levels in the control group (Fig. 3). N-acetylation of amino acids may be an indicator of protein turnover; therefore, the finding of N-acetylalanine and N-acetylcarnosine among the top candidates for GFR-related metabolites may reflect protein breakdown due to kidney damage associated with CKD. Pseudouridine is a derivative of uridine and a modified nucleoside found in RNA, although it is not currently clear how it is related to GFR. Interestingly, pseudouridine may be an ideal new biomarker to be considered in eGFR calculations because it has been repeatedly identified in studies in different ethnic groups. These studies include an investigation by Sekula and Coresh [13.27], which used samples from European and North American populations, and our study, which used samples from the Chinese population.

The random forest analysis of our study data also identified several metabolites that have not been previously reported in plasma samples. Hydroxyasparagine, also known as β-hydroxyasparagine, was ranked first in the analysis of the four groups with different mGFRs and ranked second in the serum sample[20]. Hydroxyasparagine usually appears in posttranslational modifications in EGF-like domains of humans and other eukaryote proteins, such as fibrillin-1 [28]. However, it is not currently clear how hydroxyasparagine could affect kidney filtration and GFR. N,N-dimethyl-proline-proline is another new metabolite that has not been reported previously but was selected by random forest analysis from our dataset and ranked fifth. Its association with mGFR is also not clear.

In addition to looking for potential markers associated with GFR, we also looked at other metabolites whose changes could be explained by different levels of nephropathy. This approach could then be used to confirm the credibility of the results of the metabolomics study. Creatine kinase catalyzes both the transfer of high-energy phosphate from ATP to creatine and the regeneration of ATP from creatine phosphate and ADP. Creatine slowly and spontaneously cyclizes into creatinine in solution and is then eliminated in the urine. Creatinine is well established as a marker of kidney filtration function. In our data, creatinine levels showed a continued increase from the control group to the mild, moderate and severe nephropathy groups. The urea cycle is essential for detoxifying and safely eliminating endogenous ammonia waste generated from amino acid catabolism. Blood urea levels would also be expected to rise with increasing nephropathy, which is exactly the case we observed in our study.

In addition to removing waste from the human body, another function of the kidney is to regulate electrolyte levels and the volume of body fluid. Therefore, derangements in molecules necessary for osmotic regulation are expected in patients with nephropathy. As shown in Fig. 3, many small molecule metabolites involved in osmotic regulation, such as inositol, myo-inositol chiro-inositol, erythronate, and trimethylamine N-oxide (TMAO), increased with reductions in mGFR. Other metabolites, such as 3-indoxyl sulfate, phenylacetylglutamine, 1-methylguanidine, and guanidinosuccinate, have been shown to be uremic toxins or metabolites that accumulate during uremia. The concentrations of these four metabolites also increased with an increased level of nephropathy.

Tryptophan, an essential aromatic amino acid, is involved in a number of metabolic pathways, including the production of the neurotransmitter serotonin, the anti-inflammatory metabolite kynurenine, and NAD + downstream of kynurenine. Our study identified changes in multiple metabolites in the tryptophan metabolic pathway, including kynurenine, kynurenate, anthranilate, and xanthurenate. All these metabolites were increased in the three nephropathy groups compared to that observed in the control group, indicating increased inflammation associated with decreased GFR. C-glycosyltryptophan is generated from a posttranslational modification of tryptophan by linking with sugar [29, 30]. Posttranslational modifications, such as carbamylation, have been associated with various chronic conditions, including CKD [31]. Studies in humans have consistently identified increased levels of C-glycosyltryptophan in people with a decreased eGFR.

Coresh et al. [13] provided proof of concept that a panel of novel metabolites can accurately estimate GFR. One of the goals of the current study was to discover novel metabolite markers related to glomerular filtration that could be used to improve the accuracy of eGFR estimates. Combining the results from the six different statistical feature screening methods [21], including SIS, Lasso, Optimal Lasso, SCAD, RRCS, and PLS, that examined the link between the identified metabolites and mGFR (Fig. 4), the top 30 metabolites identified in all six methods also included several of the metabolites identified in the random forest analysis (Fig. 2). After reviewing the two lists, we firmly believe that some metabolites are worth investigating for their potential ability to measure eGFR. These promising metabolites are hydroxyaparagine, pseudouridine, C-glycosyltryptophan, erythronate, N-acetylalanine, and 7-methylguanidine. Some of these metabolites have already been reported in previous studies. For example, Coresh et al. [13] proposed that pseudouridine, acetylthreonine, myoinositol, phenylacetylglutamine, and tryptophan were novel filtration biomarkers that could be used to calculate eGFR. In our study, pseudouridine was also selected as one of the top markers by random forest analysis (Fig. 1). However, the other four markers reported by Coresh et al. [13] were not identified in our study. Nevertheless, many of the potential markers selected by the random forest analysis in our study and those reported previously share similar metabolic pathways, such as acetylated amino acids (acetylserine in our study and acetylthreonine in Coresh’s study) and tryptophan metabolism (C-glycosyltryptophan in our study and tryptophan in Coresh’s study).

Interestingly, we found that creatinine was included in the top 10 important metabolites, ranked number 6 in our overall ranking using the 6 methods and was selected by at least 4 methods. This demonstrates that creatinine is still an important irreplaceable biomarker for determining GFR. Future studies will need to include more cohort individuals to validate the discovered biomarkers for targeted assays, develop rapid and sensitive detection methods and point-of-care testing (POCT) products for key biomarkers of chronic kidney disease, and utilize the potential 3–5 novel biomarkers to develop more accurate methods for GFR estimation (panel biomarkers). Advancing laboratory techniques are needed to develop, and allow the concomitant analysis of multiple biomarkers.

## Limitations of the study

In our study, many xenobiotics and metabolized xenobiotics, as well as some therapeutic drugs, were detected in plasma samples. These exogenous metabolites could have significant systemic effects on metabolism and were confounding factors in the data analysis. Future investigations will need to include more individuals in the study cohort to validate the biomarkers identified in our study, with the combined use of metabolite levels having the potential to improve estimations of GFR.

## Conclusions

This study first used random forest analysis and well-known feature selection statistical models to identify some potential glomerular filtration-related biomarkers that correlated with mGFR. These biomarkers included hydroxyaparagine, pseudouridine, C-glycosyltryptophan, erythronate, N-acetylalanine, and 7-methylguanidine. Pseudouridine has potential as an ideal novel biomarker for calculating eGFR because it appears to be independent of ethnicity. In addition, several selected metabolites, such as hydroxyasparagine and N,N-dimethyl-proline-proline, are unique metabolites that have not been reported previously. In contrast, 1,5-anhydroglucitol (1,5-AG) decreases with impaired renal function. These novel potential metabolites provide more insight into the underlying pathophysiologic processes that may contribute to the progression of CKD, lead to improvements in the estimation of GFR and provide potential therapeutic targets to improve kidney function.

## Data Availability

The datasets used and analyzed during the current study are available from the corresponding author on reasonable request.

## Abbreviations

CKD: Chronic kidney disease
ESRD: End-Stage Renal Disease
GFR: Glomerular filtration rate
mGFR: Measured glomerular filtration rate
UPLC‒MS/MS: Ultrahigh-performance liquid chromatography-tandem mass spectrometry
1,5-AG: 1,5-anhydroglucitol
eGFR: Estimated glomerular filtration rate
PCA: Principal Components Analysis
LIMS: Laboratory Information Management System
IDO: indoleamine 2,3-dioxygenase; TDO: tryptophan 2,3-dioxygenase
TMAO: Trimethylamine N-oxide
ATP: Adenosine triphosphate
ADP: Adenosine diphosphate
DHEA − S: Dehydroepiandrosterone sulfate
LASSO: Least absolute shrinkage and selection operator
Opt-Lasso: Optimal least absolute shrinkage and selection operator
SCAD: Smoothly clipped absolute deviations
ISIS: Iterative sure independent screening
RRCS: Robust rank correlation-based screening
PLS: Partial least squares
